# Myometrial Transcriptional Signatures of Human Parturition

**DOI:** 10.3389/fgene.2019.00185

**Published:** 2019-04-01

**Authors:** Zachary Stanfield, Pei F. Lai, Kaiyu Lei, Mark R. Johnson, Andrew M. Blanks, Roberto Romero, Mark R. Chance, Sam Mesiano, Mehmet Koyutürk

**Affiliations:** ^1^Systems Biology and Bioinformatics Program, Case Western Reserve University, Cleveland, OH, United States; ^2^Department of Nutrition, Case Western Reserve University, Cleveland, OH, United States; ^3^Imperial College Parturition Research Group, Department of Obstetrics and Gynecology, Imperial College School of Medicine, Chelsea and Westminster Hospital, London, United Kingdom; ^4^BGI Clinical Laboratories (Shenzhen) Co., Ltd., Shenzhen, China; ^5^Imperial College Parturition Research Group, Institute of Reproductive and Developmental Biology, London, United Kingdom; ^6^Cell and Developmental Biology, Clinical Sciences Research Laboratory, Division of Biomedical Sciences, Warwick Medical School, Coventry, United Kingdom; ^7^Perinatology Research Branch, NICHD, NIH, United States Department of Health and Human Services, Detroit, MI, United States; ^8^Department of Obstetrics and Gynecology, Wayne State University School of Medicine, Detroit, MI, United States; ^9^Center for Molecular Medicine and Genetics, Wayne State University School of Medicine, Detroit, MI, United States; ^10^Center for Proteomics and Bioinformatics, Case Western Reserve University, Cleveland, OH, United States; ^11^Case Comprehensive Cancer Center, Case Western Reserve University, Cleveland, OH, United States; ^12^Department of Reproductive Biology, Case Western Reserve University, Cleveland, OH, United States; ^13^Department of Obstetrics and Gynecology, University Hospitals of Cleveland, Case Western Reserve University, Cleveland, OH, United States; ^14^Department of Electrical Engineering and Computer Science, Case School of Engineering, Case Western Reserve University, Cleveland, OH, United States

**Keywords:** parturition, myometrium, gene expression networks, classification, inflammation

## Abstract

The process of parturition involves the transformation of the quiescent myometrium (uterine smooth muscle) to the highly contractile laboring state. This is thought to be driven by changes in gene expression in myometrial cells. Despite the existence of multiple myometrial gene expression studies, the transcriptional programs that initiate labor are not known. Here, we integrated three transcriptome datasets, one novel (NCBI Gene Expression Ominibus: GSE80172) and two existing, to characterize the gene expression changes in myometrium associated with the onset of labor at term. Computational analyses including classification, singular value decomposition, pathway enrichment, and network inference were applied to individual and combined datasets. Outcomes across studies were integrated with multiple protein and pathway databases to build a myometrial parturition signaling network. A high-confidence (significant across all studies) set of 126 labor genes were identified and machine learning models exhibited high reproducibility between studies. Labor signatures included both known (interleukins, cytokines) and unknown (apoptosis, *MYC*, cell proliferation/differentiation) pathways while cyclic AMP signaling and muscle relaxation were associated with non-labor. These signatures accurately classified and characterized the stages of labor. The data-derived parturition signaling networks provide new genes/signaling interactions to understand phenotype-specific processes and aid in future studies of parturition.

## Introduction

Parturition, the process of birth, involves dramatic changes, collectively referred to as labor, in the uterine tissues. The changes include the weakening and rupture of the fetal membranes, softening and dilation of the uterine cervix to open the gateway for birth, and activation of the myometrium (uterine smooth muscle) such that it contracts forcefully and rhythmically to become the engine for birth. Transition of the myometrium from quiescence to the highly contractile labor state is thought to be controlled at the transcriptional level through changes in the expression of specific genes whose products increase contractibility and excitability. High-dimensional transcriptome profiling technologies provide an opportunity to examine the gene expression landscape within laboring and non-laboring myometrium to identify the gene sets controlling labor. This approach allows unbiased discovery of regulatory pathways and mechanisms associated with parturition.

Transcriptional differences between laboring and non-laboring human myometrium has been examined by multiple studies in the last two decades (see Breuiller-Fouche and Germain, 2006). Early studies examined a predefined gene set or performed functional genomics via smaller expression arrays (Aguan and Carvajal, 2000; Chan et al., 2002; Charpigny et al., 2003). Over the last decade, microarray (Esplin et al., 2005; Havelock et al., 2005; Bollapragada et al., 2009; Mittal et al., 2010; Weiner et al., 2010; Sharp et al., 2016), and more recently RNA sequencing (RNA-seq) (Chan et al., 2014) platforms were used to determine the extent of expression of thousands of genes in samples of term myometrium obtained at the time of cesarean section delivery performed in women with no clinical signs of labor or from women in active labor. These studies identified multiple differentially expressed genes between laboring and non-laboring myometrium, especially with technologies that allowed greater coverage of the transcriptome. Genes with previous ties to parturition (e.g. *PTGS2*, *IL8*) were validated in multiple studies using various experimental techniques. On aggregate the data showed that labor is associated with inflammatory signals, including genes/pathways related to cytokine signaling, chemotaxis, and immune response, and to a lesser extent, genes/pathways associated with non-labor, such as smooth muscle-related processes and cell adhesion. However, despite the generation of numerous comparable myometrium transcriptome datasets, a reproducible and reliable transcriptional signature and signaling network for human labor has not been identified and comparison of the variability and consistency between larger recent studies is lacking.

**FIGURE 1:**
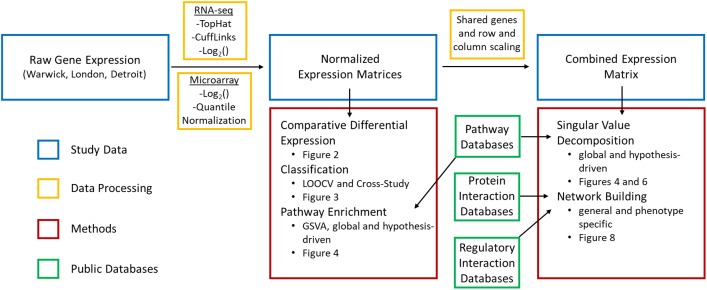
Data and methods used in bioinformatics pipeline. Raw gene expression was processed in multiple steps and combined with curated information from public databases to carry out supervised and unsupervised approaches to identify transcriptional signatures, assess their reproducibility, and identify key signaling pathways and interactions involved in parturition.

Motivated by these considerations, we performed an integrated analysis to comprehensively identify the core genes, transcriptional regulatory networks, and biological pathways involved in the transition of the term myometrium from the quiescent to the laboring state (Figure 1). To do this, we utilized two existing transcriptome datasets (Mittal et al., 2010; Chan et al., 2014) and a new dataset from RNA-seq analysis of myometrium collected from cesarean section deliveries performed at term before the onset of active labor and performed in women experiencing early (cervical dilation < 3 cm) and late labor (cervical dilation > 3 cm). A series of supervised and unsupervised computational techniques was used to extract myometrial gene expression signatures involved in the onset of labor. First, standard differential expression analysis was applied to obtain a high-level view of the discrepancies in gene expression across phenotypes and identify a high-confidence (*P* < 0.05, FC > 1.5 in all datasets) set of genes whose expression level distinguishes labor from non-labor (including previously implicated genes). We then used two machine learning algorithms to train and test gene-expression based classifiers for predicting labor. This approach also enabled the assessment of within-study sample quality and consistency as well as cross-study agreement and sample group variability. To further enhance the gene signatures identified at the first step, we used the learned model coefficients to identify the transcriptional signatures that are most predictive of phenotype for each dataset.

Unsupervised analyses were also applied to investigate whether regulatory patterns extracted from integrated gene expression data contain information regarding phenotype. For this purpose, we applied singular value decomposition (SVD) to the combined expression matrix of all studies to identify dominant patterns of expression, the genes that participate in the patterns, and the activity of the patterns in samples across studies. Overall our analyses showed the presence of a robust transcriptional signature of labor in term myometrium, both when datasets were examined individually and together. This signature was also observed at the pathway level where consistent enrichment was seen for pathways associated with labor [tumor necrosis factor alpha (*TNFA)* signaling via *NF-KB*, *MTORC1* signaling, and inflammatory response] and non-labor [vascular smooth muscle contraction, progesterone response, and cyclic AMP (cAMP) signaling]. Insights into the consistency of each dataset and sample were also obtained as well as the variability within laboring and non-laboring clinical tissue samples. Finally, we integrated our observed gene and pathway signatures with existing network information to build signaling networks of parturition and validated the networks with knowledge from the literature.

## Results

### Differential Gene Expression in Quiescent and Laboring Myometrium

Differential gene expression analysis on three transcription studies (Table 1; see Materials and Methods) was used to assess the transcriptional landscape of quiescent and laboring myometrium. The transcriptome datasets comprised two published high-quality studies (Mittal et al., 2010; Chan et al., 2014). The Chan et al. (2014) dataset is from a RNA-seq analysis of non-labor (NL; *n* = 5) and in labor (IL; *n* = 5) term myometrium collected from patients in Warwick, United Kingdom. This dataset will be designated Warwick (W)-NL and W-IL. The Mittal et al. dataset is from a microarray analysis of NL (*n* = 20) and IL (*n* = 19) term myometrium patients in Detroit, MI, United States. This dataset will be designated Detroit (D)-NL and D-IL. We performed a RNA-seq study using myometrium collected from 22 women at term cesarean section delivery undertaken at the Chelsea and Westminster Hospital, London, United Kingdom. This study, designated London (L), included L-NL (*n* = 8) and L-IL (*n* = 14) myometrium. The L-IL tissue was further stratified into early (Ea) stages of active labor (L-ILEa: defined as contractions with cervical dilation < 3 cm, *n* = 8) and during established (Es) labor (L-ILEs: defined as contractions with cervical dilation > 3 cm, *n* = 6) (see Materials and Methods for more detail regarding sample collection and RNA preparation and measurement.).

**Table 1:** Myometrial tissue gene expression studies analyzed in this work.

Study	Platform	Sample groups	Group size	Clinical definition of labor
Warwick	RNA-seq	Non-labor (W-IL) In-labor (W-IL)	*N* = 5 *N* = 5	Regular contractions (<3 min apart), membrane rupture, and cervical dilation (>2 cm)
London	RNA-seq	Non-labor (L-NL) Early-labor (L-ILEa) Established-labor (L-ILEs)	*N* = 8 *N* = 8 *N* = 6	Cervical dilation < 3cm (L-ILEa) Cervical dilation > 3cm (L-ILEs)
Detroit	Microarray	Non-labor (W-NL) In-labor (W-IL)	*N* = 20 *N* = 19	Contractions (<10 min apart) and cervical dilation requiring hospitalization

*Three gene expression datasets collected from publicly available sources or collaborators are shown. Sample groups and names are consistent with the acronyms listed in column three throughout the paper. Clinical definitions of labor represent how each sample was called phenotypically, based on the patient’s clinical presentation at the time of cesarean section, as chosen by the researchers collecting the samples.*

The RNA-seq datasets were analyzed using the TopHat and Cufflinks software and the microarray dataset was processed using the method described in the parent publication by Mittal et al. (2010) which applies log_2_ transformation and quantile normalization followed by empirical Bayes statistical tests on linear models fit to the chip probes (see Materials and Methods). Genes having a *p*-value (adjusted using the Benjamini Hochberg procedure for multiple hypothesis testing) less than 0.05 and a fold change greater than 1.5 were deemed significant.

For the London dataset, significant differential expression was detected for 417 genes between L-NL and L-ILEa (397 up and 20 down in L-ILEa), 547 between L-NL and L-ILEs (440 up and 107 down in L-ILEs), and, surprisingly, none between L-ILEs and L-ILEa. Initial analyses detected no significant difference in the transcriptomes of L-ILEa and L-ILEs, which suggests that gene expression changes associated with the onset of labor persist through early and late stages of the parturition process. This outcome also indicates that labor associated changes in myometrial gene expression occur early in the labor process and persist.

Our analysis of the Warwick dataset identified 2468 genes differentially expressed (1227 increased and 1241 decreased based on mRNA abundance; *P* < 0.05 and fold change > 1.5) in W-IL compared with W-NL myometrium. In the parent publication Chan et al. (2014) used the results of three separate software packages to calculate differential expression to improve confidence. Our result is comparable to the number obtained by one of these packages, edgeR. Our analysis of the Detroit dataset identified 463 genes that were differentially expressed (267 up and 196) in D-IL compared with D-NL myometrium. This is highly consistent with the number obtained in the parent publication (471 in Mittal et al., 2010) Figure 2 shows the overlap of differentially expressed genes across the four within-study phenotype comparisons. For the Detroit and London comparisons a similar number of genes were differentially expressed between the NL and IL tissues, with the majority of genes up-regulated in the IL myometrium while the Warwick comparison showed a very strong change in expression with almost five times as many differentially expressed genes compared to the other datasets. Pairwise comparisons between all labor conditions in the three studies identified 358 shared genes for Warwick and Detroit, 339 for Warwick and London (L-ILEa), 410 for Warwick and London (L-ILEs), 156 for Detroit and London (L-ILEa), 180 for Detroit and London (L-ILEs), and 282 for L-ILEa and L-ILEs.

**FIGURE 2:**
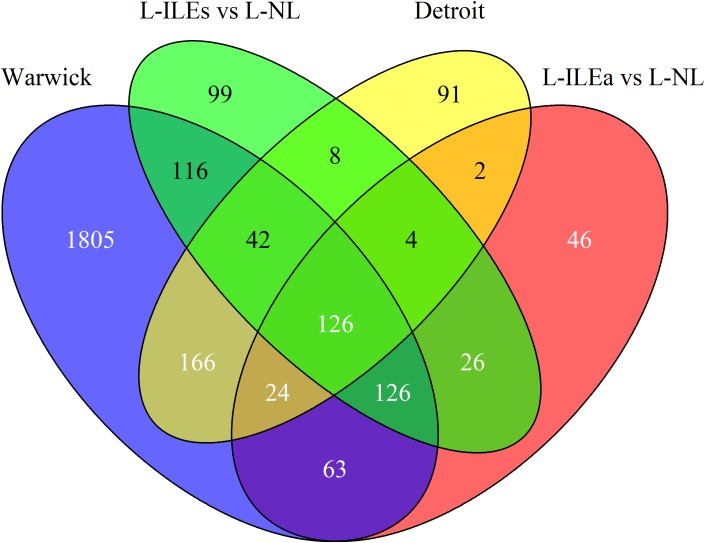
Venn diagram of differential expression in Warwick, Detroit, and London. Differentially expressed genes between non-labor and in-labor sample groups for each dataset are compared to calculate similarity across studies.

We identified 126 genes (124 increased and 2 decreased) that were differentially expressed in all comparisons [see Supplementary Table S1 for a list of these genes with their median log_2_(fold change) and median adjusted *p*-value]. Thus, we can have high confidence in these 126 genes as being true positives for identifying biological processes that likely initiate and/or maintain the labor phenotype. Using these high-confidence genes, we performed pathway enrichment analysis using the Kyoto Encyclopedia of Genes and Genomes (KEGG) pathways with the online tool EnrichR. This analysis identified multiple significant pathways including *TNF* signaling (adjusted *p*-value = 1.2e-9), cytokine-cytokine receptor interaction (4.6e-9), *Jak-STAT* signaling (2.4e-7), *NOD*-like receptor signaling (2.3e-4), and chemokine signaling (8.9e-4). These results indicate a strong inflammatory component, which is supported by previous studies and suggests that a significant portion of the transcriptional changes shared by all studies represents an increase of inflammatory signaling with the onset of labor.

### Within-Study Classification

A machine learning approach was used to identify the best set of genes that distinguish labor from non-labor. This approach was also useful in assessing the consistency of samples within and across datasets. For this purpose, we employed two sparse regression-based classification approaches that are well-established in the area of machine learning: (1) least absolute shrinkage and selection operator (LASSO), and (2) elastic net (EN).

Least absolute shrinkage and selection operator utilizes a regularization term, the L1 norm of model coefficients, penalizing models that contain more features (Tibshirani, 1996). This results in more parsimonious predictive models, which allows for easier interpretation of the final classifiers (i.e., small number of genes in the model). EN is an extension of LASSO in that it includes an additional penalty term in the form of the L2-norm of the model weights (Zou and Hastie, 2005). This serves to avoid shortcomings of LASSO, such as its restriction to choosing at most n (the number of samples) features in its model and tendency to only select one feature from sets of highly correlated features. With this additional term in the objective function, EN models typically include more features (genes) than the models built by LASSO. Here, we employed both EN and LASSO to obtain the smallest set of genes that can predict labor (LASSO), while also allowing models with a larger number of genes (EN), of which some may exhibit highly similar patterns of expression. The latter can result in models with multiple genes that show similar differential expression across the IL and NL tissues, all of which can be of interest for our analysis. Besides providing a means to identify genes that can serve as predictive features, use of classifiers also provides a way to assess the consistency of sample groups within and across datasets.

**FIGURE 3:**
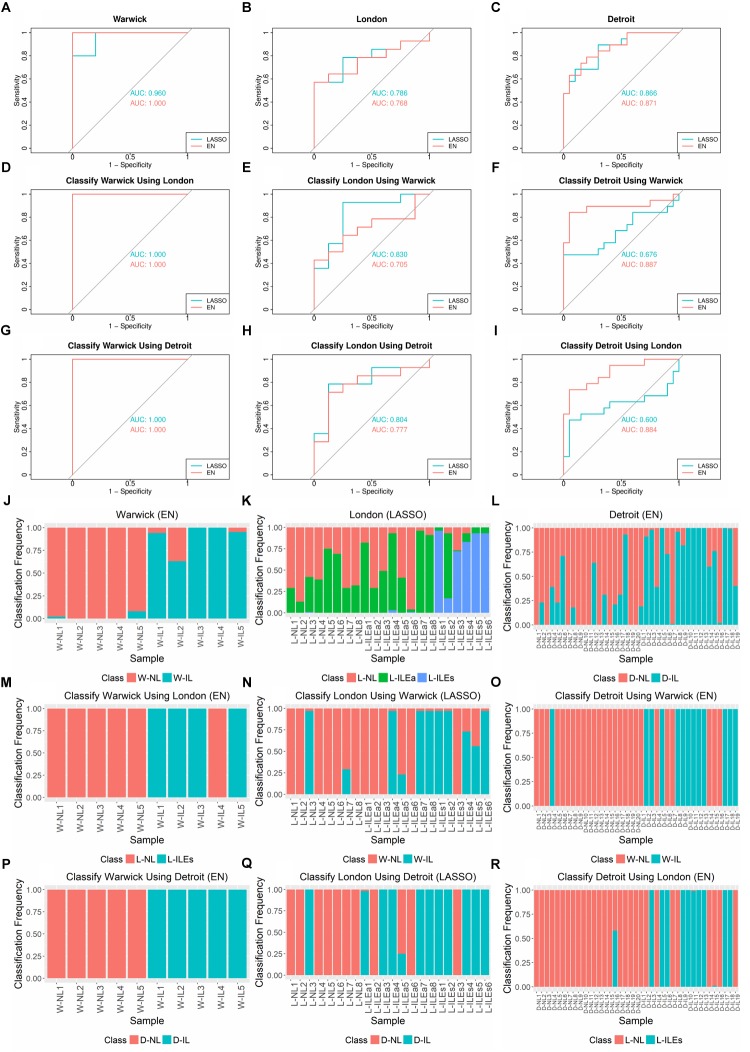
AUCs, ROC Curves, and sample classification frequencies for K-fold cross-validation and cross-study classification tasks on tissue gene expression studies using LASSO and EN. **(A–C)** K-fold cross validation (CV) for each dataset (*k* = 3 for Warwick, 5 for London and Detroit). **(D–I)** Classification of one dataset with training performed on another dataset. LASSO and EN were implemented using the *glmnet* package in R. **(J–R)** Corresponding sample class frequencies for the classification tasks shown in **(A–I)**, specifically using the results from the top performing algorithm, based on AUC. For all prediction tasks, the early labor (L-ILEa) and established labor (L-ILEs) samples were grouped together as a single laboring sample group except for sample classification frequency for 5-fold CV on London, where the three sample groups were kept separate in order to assess how the new initial labor group was classified. All results shown were obtained using 100 runs of training and testing. Sample names in **(J–R)** follow the naming convention in Table 1.

Using the R *glmnet* package, we performed 100 runs of *k*-fold cross validation (CV) to train and test the models. Nested cross validation was used to optimize model parameters. Due to the varying number of samples in each study, we set *k* = 3 for the Warwick dataset and *k* = 5 for the London and Detroit datasets. The performance of LASSO and EN in classifying the samples from each study was assessed by receiver operating characteristic (ROC) and area under the ROC curve (AUC) (Figure 3A–C). Varying levels of performance were achieved across the datasets, with AUC being near perfect (>99%) for Warwick, good for Detroit (∼90%), and fair for London (∼75%). In these experiments, for the London dataset, we grouped the L-ILEa and L-ILEs samples into one L-IL group so that labor was represented as a single phenotype for the calculation of AUC.

We also examined the frequency with which each sample was classified into each phenotype over the 100 runs of training and testing (Figure 3J–L). Based on these results, we observed several interesting patterns. First, for classification frequency calculations, we ungrouped the two L-IL sample types to assess how they separated based on the built classification models. The L-ILEa class appeared to be more variable and difficult to classify, seeming to have signatures partially represented in the L-NL and L-ILEs group. Second, classification of the non-labor samples was highly accurate across all studies, suggesting the presence of a robust gene expression profile characteristic of the quiescent pre-labor myometrium. Finally, there were samples with a classification distribution that was highly contradictory to their given phenotype (i.e., L-ILEa6, D-NL18, D-IL7, and D-IL16).

To gather information on the genes that were used to achieve the classification results, we examined the features selected in the models built during training. For the Warwick dataset, 147 genes were selected in at least one EN model, and 22 of the genes were selected in all 100 EN models (Supplementary Table S2). For the London dataset, to further observe how the L-ILEa group was classified, we did not combine the L-ILEa and L-ILEs samples for this sample classification frequency calculation. For this reason, there are three classes in the classification task, meaning it is a multinomial problem. In this case, when assessing models and the gene coefficients, there are genes associated with each class (these are the genes that best separate each class from the other two). The results were 7 unique genes for the L-NL class, 12 for the L-ILEa class, and 12 for the L-ILEs class in at least one of the 100 LASSO models (Supplementary Tables S2–S4). For the Detroit dataset, 98 genes were selected in at least one of the 100 EN models, 32 of which were selected in all models (Supplementary Table S5). The models were approximately evenly divided between genes increased in IL samples and those decreased in NL samples across the three studies (Supplementary Tables include model genes and average coefficients).

Next, we compared the genes selected by the classification models to those that were identified via differential expression analysis 0. 97 of 147 Warwick model genes were significantly differentially expressed in the Warwick dataset, two of these were high-confidence genes that were differentially expressed in all comparisons (total of 126). The two high-confidence genes were also among the 22 genes that were selected in all 100 Warwick models. In contrast, 19 of the 98 Detroit model genes were significantly differentially expressed in the Detroit dataset with only one being a high-confidence gene. Of the 32 genes that were selected in all Detroit models, 12 were differentially expressed in the Detroit dataset, and one was a high-confidence gene. Finally, 2 of the 7 L-NL, 1 of the 12 L-ILEa, and none of the L-ILEs genes for the London models were significantly differentially expressed in the London dataset. The number of significant genes in the models for each dataset correlate with the accuracy of classification (e.g. Warwick has the highest percentage of incorporated significantly called genes and the highest AUC).

Interestingly, models built on different datasets shared only two genes, *MITF* and *CLK2*, which were found in respectively 100 and 87 of the Warwick EN models and in respectively 96 and 18 of the Detroit EN models. However, neither of these two genes were among the 126 high-confidence genes that were identified via differential expression analysis. It has been shown that MITF is involved in the suppression of *IL-8*, a classic inflammatory gene, in the cervix during pregnancy (Li et al., 2010). This agrees with the classification models derived from the Warwick and Detroit datasets as MITF has a negative model coefficient (higher expression in non-labor group). No direct associates between *CLK2* and parturition were observed in the literature, however, data from the human protein atlas shows that *CLK2* exhibits a higher expression in leukocytes compared to smooth muscle (Uhlén et al., 2015). Therefore, the positive model coefficient observed for *CLK2* may represent leukocytes (accompanying increased inflammation) in the laboring tissue.

### Cross-Study Classification

The overlap between models learned from different studies is low in terms of gene content, although all models represent the difference between myometrial quiescence and labor. Based on this observation, we hypothesized that the models learned using each dataset may be capturing a different aspect of the signaling processes that underlie labor. To test this hypothesis, we classified the samples in each dataset by using models learned from each of the other two datasets. Note that training on London data was done only using the L-NL and L-ILEs sample groups to be consistent with Warwick and Detroit protocols. As in the previous section, LASSO and EN were used for classification, nested k-fold cross validation was used to optimize model parameters, and 100 iterations of training and testing were performed.

The results of cross-study classification are shown in Figure 3D–I. Since there are three studies and training on each dataset was followed by testing on the remaining two datasets, there were a total of six cross-study classification tasks. Assessing the average performance in each of the six classification tasks, we observed that models learned on one dataset were overall successful in classifying samples from a separate dataset, with AUCs ranging from 0.676 to 1 and a median AUC of 0.817. Classification of the Warwick samples proved to be a simple task for the models built using each of the London and Detroit datasets. This is likely because the Warwick dataset has a larger set (∼2,000 more than the other two studies) of differentially expressed genes between the laboring and non-laboring groups. We also observed that LASSO models performed better for classification of the London samples relative to EN. This could be due to the fact that many genes in a model may create additional uncertainty for classifying the more volatile L-ILEa samples. Another interesting result is that models derived from the Warwick and Detroit datasets produced similar performance on the London dataset despite the fact that Detroit has almost four times as many samples.

As was done for the cross-validation analysis, we also computed the sample classification frequencies for the cross-classification tasks (Figure 3M–R). Namely, we assessed the confidence with which one dataset correctly called the phenotype of a given sample from another dataset. Samples not always classified into the same group may have a weaker signature representation than others. We found that the samples highly misclassified in the cross-validation analysis (e.g. L-ILEa6, D-IL7, and D-IL16) showed the same pattern when classified using other datasets.

Overall, the cross-study analysis indicates a robust transcriptional signature differentiating NL from IL across the three datasets. Namely, the performance in all classification tasks ranged from moderately accurate (AUC ≈ 0.7) to highly accurate (AUC ≈ 1). This is fairly impressive for a gene expression based classifier given that these types of classifiers often fail to generate reproducible models due to experimental noise, heterogeneity of samples, and the limitations of mRNA-level expression in capturing changes in protein activity and function. This observation suggests that transcriptional regulation may play a key role in the transition of myometrial tissue from quiescence to the laboring phenotype.

### Identification of Latent Transcriptional Regulation Patterns via Singular Value Decomposition

To understand underlying patterns of gene expression associated with labor, we used SVD, an unsupervised, dimensionality reduction and pattern identification method that has been previously used to identify latent transcriptional regulation patterns in gene expression data (Alter et al., 2000, 2003; Tomfohr et al., 2005).

We performed SVD on the expression matrix containing all 71 samples (10 Warwick + 22 London + 39 Detroit) and 12,622 genes (those with measured expression in all studies). For a given gene expression matrix *M*, SVD decomposes this matrix into three parts: a pair of singular vectors, which can be interpreted as an eigen-sample and eigen-gene pair, and a relative strength value called a singular value. An eigen-gene represents a subset of genes with a given, similar pattern of expression. The eigen-gene then contains a value for each sample (*n* = 71) indicating how the subset of genes is expressed in each of them. An eigen-sample represents a subset of samples with a given, similar pattern of activity across the corresponding eigen-gene. The eigen-sample then contains a value for each gene (12,622) indicating the activity of the subset of samples for a set of genes. The corresponding singular value indicates how well represented or how common that pattern of expression is within a gene expression matrix (Alter et al., 2000). Therefore, we employed SVD to determine the dominant signals or expression programs within the combined tissue expression matrix, identify the genes that had the strongest contribution to the observed patterns, and visualize the stratification of genes and samples in a reduced space.

**FIGURE 4:**
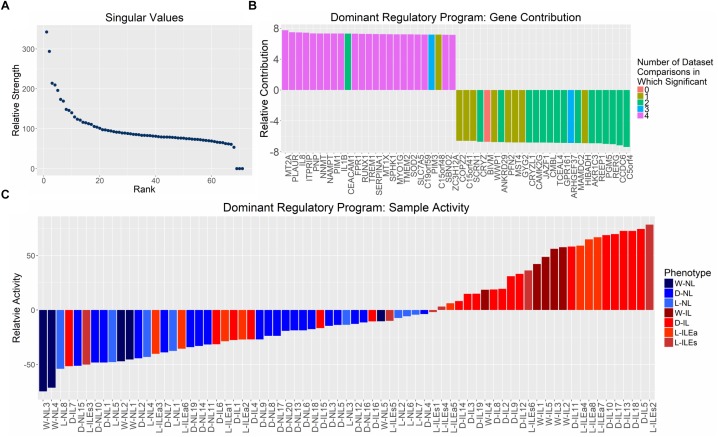
Distribution of singular values and examination of genes and samples in the space spanned by the first singular vector from SVD analysis on aggregated gene expression matrix. **(A)** Singular values calculated by performing SVD on the normalized, study-combined expression matrix (12,622 genes by 71 samples). **(B)** Mapping of the top 25 positively and negatively valued genes in the space represented by the first right singular vector (eigen-gene). Bars representing genes are colored based on its differential expression in each of the four differential expression analyses (shown in Figure 1; i.e., purple bars indicate genes in the 126 set of high-confidence genes). **(C)** Mapping of all tissue samples to the first left singular vector (eigen-sample), sorted by mapping value. Bars representing samples are colored based on their clinically defined phenotype (blue = non-laboring and red = laboring). Sample name abbreviations in **(C)** follow Table 1.

Examination of the singular values of the combined gene expression matrix indicate one, potentially two, important patterns (Figure 4A). This first singular vector was used for further analysis as it captures the main component of variation in the expression matrix and can be thought of as the dominant regulatory program. The dominant pattern among samples, obtained from the first eigen-gene, shows a clear separation of NL and IL samples with the former having negative values in the singular vector and the latter having (mostly) positive values (Figure 4C). The strength of this state of differential expression in each sample is determined by observing the magnitude of each sample’s loading in the first eigen-gene. Based on these values, varying levels of pattern consistency across studies and within phenotypes were identified. Figure 4C suggests that IL samples are more heterogeneous or potentially more difficult to call phenotypically (e.g., L-ILEs3 and D-IL7 are highly inconsistent) than non-labor samples, which always have the same loading on the dominant singular vector. Based on these observations, we can assert that the dominant eigen-gene of the combined expression pattern represents the separation of IL and NL samples.

We examined the most dominant eigen-sample to identify the genes that contribute most to this singular vector, since these genes most accurately represent the labor phenotype. The 25 genes with the largest positive and negative values on the first eigen-sample are shown in Figure 4B. To provide a context for the correspondence between differential expression and this latent transcriptional regulation pattern, each gene is represented as a colored bar where the color is based on the number of study comparisons in which its expression pattern showed differential expression between phenotypes (e.g., purple bars indicate genes that were part of the 126 high-confidence genes from Figure 2). The positively contributing genes in Figure 4B were associated with higher expression in samples having a positive loading, mainly the IL samples. These genes strongly represented inflammatory processes, which has been shown in previous studies, containing genes such as *IL-8*, *RUNX1*, and *IL-1β*. The negatively contributing genes were more highly expressed in the NL samples and were not as well-replicated across all studies, but had comparable magnitudes of pattern contribution when compared to the positively loaded genes. One of these genes, *GPR161* increases intracellular cAMP and promotes protein kinase A (*PKA)*-dependent processes. This aligns with the hypothesis that the cAMP*/PKA* pathway plays a negative role in parturition by promoting relaxation of the myometrium (Yuan and Bernal, 2007). Genes including *JAZF1* and *TCEAL4* are involved in transcriptional regulation while little is known about the function of *MAMDC2* and *BIVM*. Further investigation of these genes may provide additional hypotheses relating to the maintenance of pregnancy.

In summary, the dominant singular vector of our combined expression matrix represents a differential expression of genes between IL and NL myometrium. We propose that SVD directly presents an unsupervised way to classify the phenotype of any myometrial sample, via gene expression, using the first singular vector.

### Global Identification of Pathways Altered During Parturition

Based on the results of our comprehensive computational experiments using classification and SVD, it is clear that IL and NL myometrium exhibit strong differences in their transcriptional profiles. To better understand and interpret the signatures obtained, we performed pathway enrichment analysis using Gene Set Variation Analysis (GSVA), with the hallmark gene sets from the molecular signatures database (MSigDB; well-defined biological states or processes created by collating gene sets from multiple sources), to each study individually. GSVA is an unsupervised method that makes use of the full expression matrix as opposed to a simple list of differentially expressed genes (e.g., over-representation) and provides a directionality, as fold change, for each pathway. This permits GSVA to determine pathway enrichment similarly to other approaches in that strong differential expression in a few pathway genes will be significant, but also in cases where there is potentially weak but highly consistent differential expression across many genes in a pathway. We chose to perform GSVA on the studies separately to make use of GSVA’s enrichment fold change output (i.e., we can assess how pathways are being changed from NL to L as opposed to simply calling them significant with no directionality). As mentioned in the Methods section, an additional normalization step was required to combine all studies (as in the SVD analysis). This normalization shifts the expression distribution, diminishing the resulting fold change values, making it difficult to determine significance using traditional methods (i.e., fold change cutoff of 1.5). We selected the hallmark gene sets from MSigDB for our analysis. Pathway enrichment in this form allows better interpretation of the strongest signal between IL and NL samples specific to each study as well as identify pathway-level consistency across all studies.

**FIGURE 5:**
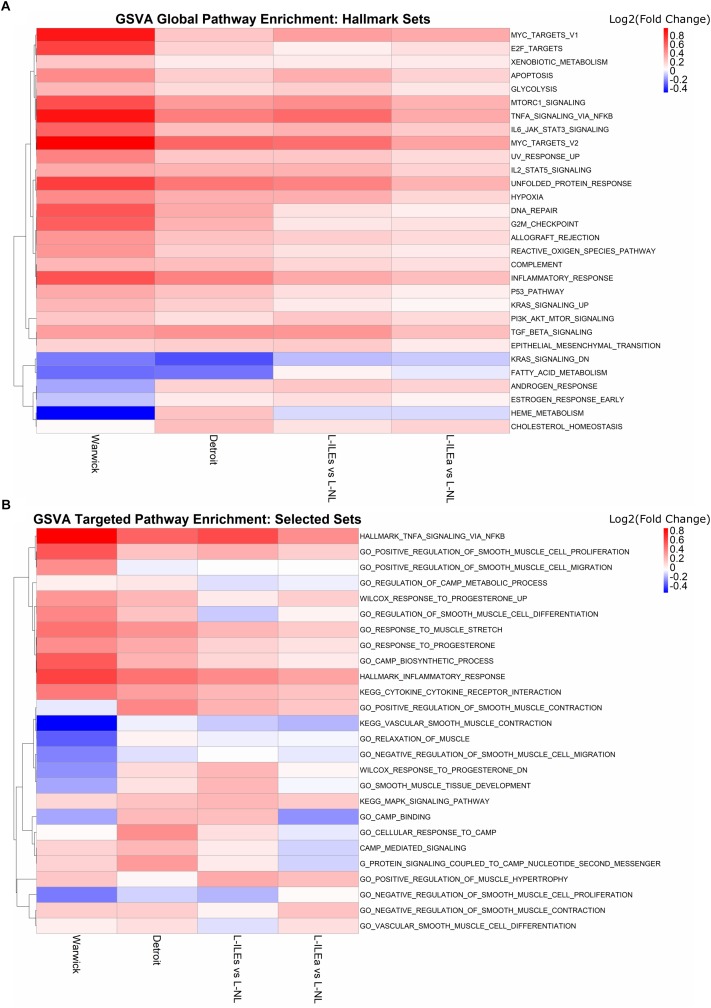
Global and hypothesis-driven GSVA pathway enrichment across all studies. **(A)** Top 30 enriched pathways (by median adjusted *p*-value) using the hallmark gene sets from MSigDB. **(B)** Enrichment results for a selected set of pathways from MSigDB related to progesterone response, smooth muscle, inflammation, and cAMP signaling. Heat map is colored by log_2_(fold change) where red means up-regulation in laboring samples and blue down-regulation. Rows (gene sets) are clustered using the complete clustering method based on correlation.

Figure 5A shows the GSVA results on the Warwick, London, and Detroit datasets, where rows (gene sets) are clustered by fold change enrichment. In the figure, pathways with higher activity in NL samples are shown in blue and pathways with higher activity in IL samples are shown in red. All three studies exhibited consistent enrichment, specifically upregulation, for multiple gene sets including *TNF*, *MYC* targets, mechanistic target of rapamycin kinase (*MTORC1*), inflammatory response, and Janus kinase (*JAK*)/signal transducers and activators of transcription (*STAT*) signaling gene sets. Note that not every pathway was significant in every study under traditional cutoffs (FC > 1.5 and adj. *p*-value < 0.05). Expression of *TNF-α* has been shown to increase during labor in the cervix, fetal membranes, and myometrium, where it stimulates arachidonic acid release, activates phospholipid metabolism, and increase the production of prostaglandins in the myometrium (see review Peltier, 2003). The *JAK/STAT* pathway is a major cellular signaling pathway that has been shown to be activated by mechanical stretch of myocytes (Pan et al., 1999). *JAK*s are also activated by cytokine ligands, including interferons and interleukins, which are known to be associated with labor (Brooks et al., 2014). Additionally, a study by Breuiller-Fouche et al. (2007) that combined results from existing studies examining labor in the myometrium showed that many of the consistently discovered genes were involved in the JAK-STAT pathway. From our results, genes involved in TNF signaling include *SOCS3, IL6, CEBPB, IL1B, BCL3, LIF, TNFAIP3, CCL2, PTGS2, SELE*, and *CXCL2*, while the genes *SOCS3, CSF3, IL6, IL4R, CSF3R, MYC, OSM, PIM1, LIF*, and *IL7R* make up the *JAK-STAT* pathway. Other pathways observed to be consistently enriched have links to parturition. Master et al. (2002) showed that *MYC* expression in the mammary glands of pregnant FVB mice decreases during pregnancy and rises just before labor and work by Deng et al. (2016) suggested an important role for *mTORC1* signaling in the control of parturition timing. Together, the results of this enrichment analysis both confirms and annotates the transcriptional signature we have seen in the above sections with specific pathways that can provide starting points for more targeted studies involving the transition of the myometrium from quiescence to labor.

### Hypothesis-Driven Signature Identification

To expand our search to pathways reported before to play a role in parturition, we selected 32 gene sets from MSigDB that were related to progesterone signaling, inflammation, and muscle relaxation. We repeated GSVA with these sets for each study (Figure 5B). As in the previous section, results are represented in terms of a colored (by GSVA fold change enrichment), clustered heat map. There are clear areas of the heat map that indicate, for multiple gene sets, differential expression and, just as importantly, fair consistency across most or all study comparisons. These sets include *TNFA* signaling via *NFKB* and inflammatory response upregulated in labor and vascular smooth muscle contraction, regulation of cAMP metabolic process, and relaxation of muscle downregulated in labor. Such results indicate the importance of these pathways in parturition.

**FIGURE 6:**
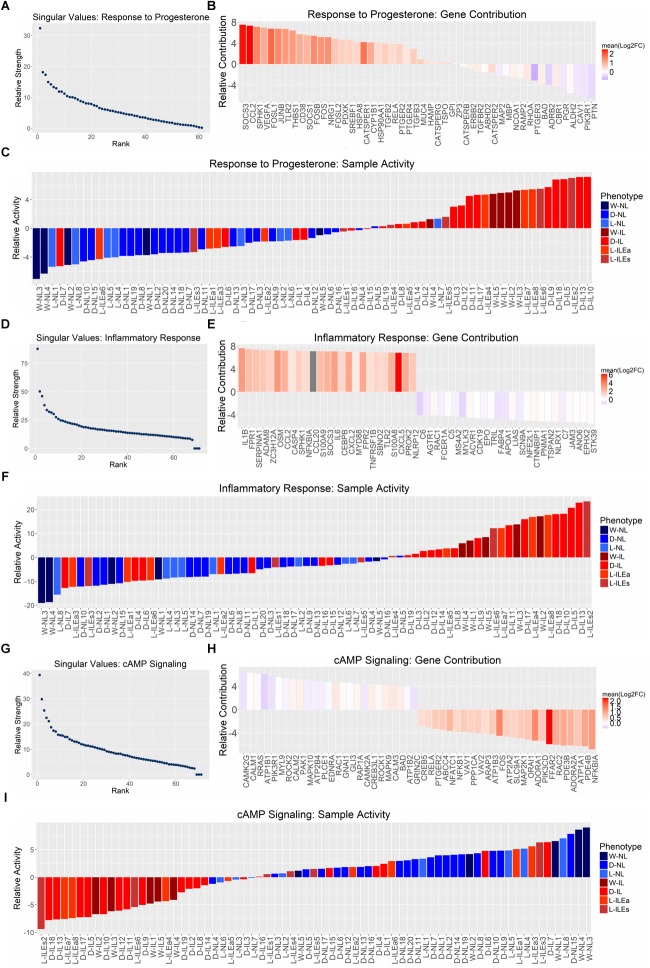
Pathway-specific SVD analysis. SVD results for the gene sets **(A)** GO response to progesterone, **(B)** hallmark inflammatory response, and **(C)** KEGG cAMP signaling pathway. All studies were combined (12,622 genes × 71 tissues) and then reduced to the genes within each gene set. **(A**,**D**,**G)** Show the distribution of singular values, **(B,E,H)** show the top 25 genes associated with each phenotype based on loading value [genes are colored by their mean log_2_(fold change) value across all four sample group comparisons (Figure 1)], and **(C,F,I)** show the gene set activity (loading value) for each sample (samples are colored based on their originally reported clinical phenotype).

Next, we applied SVD to genes involved in signaling by progesterone, inflammation, and cAMP. The most representative gene sets selected for this analysis were Gene Ontology’s response to progesterone, MSigDB’s hallmark inflammatory response, and KEGG’s cAMP signaling. Each gene expression study was reduced to the genes in one of the sets that had normalized measures of expression in all studies. The pathway-specific expression matrices were then concatenated and subjected to SVD. Results indicate that all three pathways exhibit differential expression between phenotypes, since the dominant regulatory program (eigen-gene and eigen-sample pair) for each gene set clearly separates the IL and NL samples (Figure 6), similar to our global SVD result.

The relative strength of the dominant regulatory programs can be compared between these three pathways by using a measure introduced in Leskovec et al. (2014) called “energy.” The square of each singular value is a measure of the amount of variation accounted for by the first eigen-gene and eigen-sample pair (Tomfohr et al., 2005). It then follows that the squared sum of all singular values represents the total variation of the data. Then the energy measure of interest here is the proportion of total variation (in the gene expression data) covered by the first singular vector pair, which can be calculated by dividing the square of the first singular value by the sum of squares of all singular values. Using this measure, the energies are 24.28% for response to progesterone, 23.26% for inflammatory response, and 15.89% for cAMP signaling. Therefore, the separation between IL and NL samples is strong for response to progesterone and inflammatory response and less so for cAMP signaling. Positive sample loadings are seen for the laboring phenotype for response to progesterone and inflammatory response while positive sample loadings for the non-laboring phenotype are seen in the case of cAMP signaling. This is consistent with our understanding that inflammatory response is pro-labor and cAMP signaling is relaxatory or an anti-labor process. The gene set response to progesterone has many inflammation-related genes due to the fact that it is anti-inflammatory (i.e., expression of these genes is decreased in response to inflammation), therefore, the samples map in the same direction as the inflammatory response SVD result. However, gene loadings can provide insights into the pro-relaxation genes that progesterone regulates. Figure 6B,E,H rank contributions of genes in these pathways, showing which genes contribute to the association of the pathway with each phenotype. Due to the strength of the inflammatory/laboring signal, the observed fold change is more consistent across studies for genes in this set than those with higher expression in the non-laboring signal. Overall, these results indicate that there is some level of differential expression for these pathways in our transcriptome studies. However, many pathways are comprised of various signaling processes. Certain genes in a pathway may be pro-quiescent and down-regulated at the onset of labor, while others in the same pathway promote labor and are up-regulated during labor. An example is cAMP signaling, which has canonical paths in various tissues that activate inflammatory-related processes (*MAPK* and *PI3K-AKT*signaling) as well as paths that activate muscle relaxation. Such pathways are clearly relevant to parturition but may have reduced significance (enrichment fold change) due to the regulation of the genes involved in these pathways in different directions.

**FIGURE 7:**
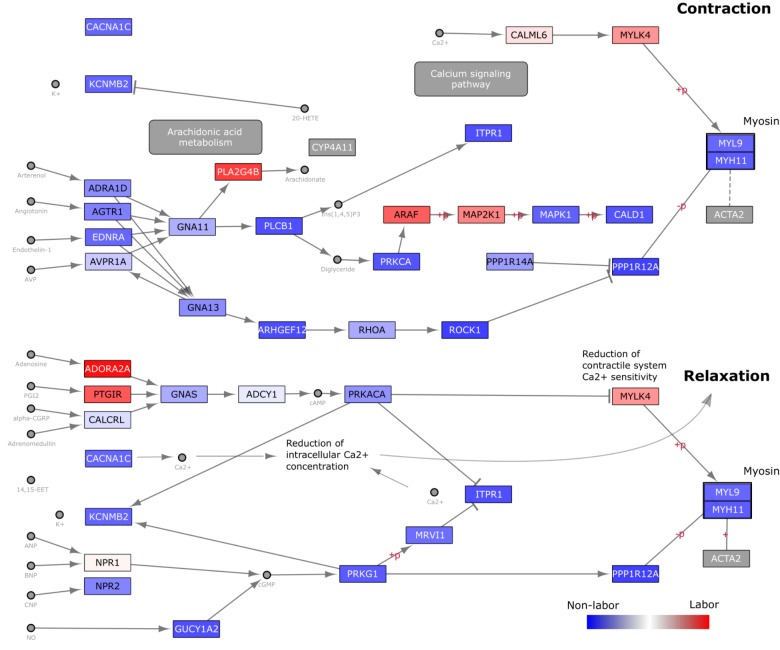
Visualization of gene expression changes on KEGG Vascular Smooth Muscle Contraction (VSMC) Pathway Diagram. The *Homo sapiens* VSMC pathway diagram was downloaded in KGML format from KEGG and loaded into Cytoscape. Nodes (genes) are colored based on loading values for the dominant singular vector from performing singular value decomposition on the reduced (to include only genes within the VSMC pathway) Warwick expression matrix. These values were directly loaded into Cytoscape as a node table and used in a continuous manner in the style tab for node coloring. Blue nodes indicate genes with higher expression in the W-NL group, red higher expression in the W-IL group, and white no differential expression.

Next, we focused on a pathway that was consistently, but not significantly, enriched in the targeted GSVA analysis: vascular smooth muscle contraction (VSMC). We overlaid gene loadings from the first eigen-sample obtained from performing SVD on this pathway (Figure 7). By analyzing a pathway in this manner, we can better understand how changes in expression may be influencing signaling paths as opposed to just knowing whether or not the expression of a group of genes is changing in one direction or another. A large majority of the genes in this pathway have higher expression in the NL samples, suggesting that a significant part of the signaling machinery for contraction is used to keep myometrial cells in a relaxed, quiescent state during pregnancy. Then, at the time of labor, a small number of important genes in specific areas of this pathway are up-regulated to achieve myometrial contraction. The labor-triggering genes are involved in initiating inflammatory response (*PLA2G4B*), *MAPK* signaling (*ARAF, MAP2K1*), and calcium-induced contraction (*CALM6, MYLK4*).

### Building a Parturition Signaling Network

The problem of reconstructing biochemical pathways is well studied, and the shortest paths approach has been shown to be an accurate solution (Croes et al., 2005; Silverbush and Sharan, 2014; Ritz et al., 2016). We used a curated signaling network constructed by Ritz et al. (2016) by integrating multiple protein interaction and pathway databases as well as Gene Ontology (GO) (see Materials and Methods). This network is directed and weighted, where weights are calculated using a Bayesian approach to assign probabilities to edges based on experimental evidence and shared GO terms (Ritz et al., 2016). Since we have identified progesterone signaling, inflammation, and cAMP signaling as important pathways based on enrichment results and prior work in the parturition field, we reduced the network by selecting the nodes and edges that are downstream of the proteins *PGR*, *IL1R1*, and *ADCY*. These three proteins are the main, upstream initiators for the three pathways of focus (see Materials and Methods for the justification of these protein choices). We call these three proteins the “source” nodes in the parturition signaling network. We then assigned weights to the nodes of the resulting network based on the broad gene expression signature derived from our global SVD analysis (i.e., the weight of a gene product in the network is the absolute value of the loading of the gene in the most dominant eigen-sample). We also chose “sink” nodes in the parturition network as the products of the top 50 genes associated with laboring and non-laboring myometrium based on the absolute values of the gene loadings. This choice of the top 50 is somewhat arbitrary, however, it ensures an equal representation from each phenotype while capturing the most significant changes at the gene expression level. This choice also allows for a relatively broad signal (as there are 100 total genes) without being too large, which would result in very dense networks. We then calculated shortest paths from each of the source nodes (the three pathway initiator proteins) to each of the sink nodes (the highly differentially expressed genes), by ensuring that the last edge in the path is an edge between a transcription factor and a target node. All nodes that lie on one such shortest path are included in the final parturition signaling network.

**FIGURE 8:**
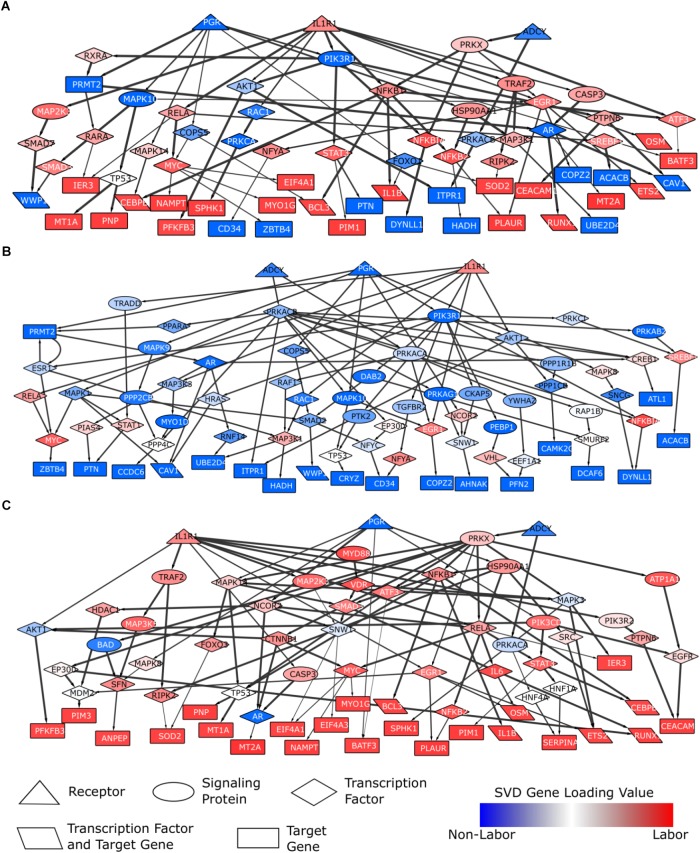
Parturition-specific signaling networks constructed using shortest paths and differential expression data. Shortest paths were calculated on a curated, directed, weighted signaling network. Shortest paths were obtained from each of the three pathway initiator proteins (*PGR*, *IL1R1*, *ADCY*; chosen to represent progesterone, inflammatory, and cAMP signaling) to each downstream target gene. Nodes in the network were weighted based on gene loadings from the dominant singular vector when performing SVD on the combined expression matrix (3 studies; 12622 genes × 71 samples). **(A)** A general parturition signaling network. Target genes consisted of the top 50 genes associated with the non-laboring and laboring sample groups (100 total input genes). Nodes were weighted such that those with strong phenotype associations had the lowest cost. Paths falling in the top 50%, in terms of lowest costs, were retained. **(B)** A parturition signaling network prior to labor (i.e., quiescence). Target genes consisted of the top 50 genes associated with the non-laboring sample groups. Nodes were weighted such that genes highly expressed in the non-laboring samples had low costs, those with roughly equal expression across the two phenotypes had a medium cost, and genes with higher expression in the laboring samples had high cost. **(C)** A parturition signaling network during labor. Target genes consisted of the top 50 genes associated with the non-laboring sample groups. Nodes were weighted opposite of the quiescent network in **(B)**. All paths were retained for the networks in **(B,C)**. Networks were visualized in Cytoscape. Node shapes are based on function/pathway location, node colors correspond to their respective weights, and edge width is proportional to the weights in the original network (thicker meaning higher confidence).

The resulting network generated from this method has 66 nodes and 91 edges (Figure 8A). The network contains four different node types: receptor, signaling protein, transcription factor, and target gene. The transcription factors and signaling proteins were selected by the shortest paths calculations. Nodes are colored based on their weight (gene loadings from the dominant regulatory program from our global SVD analysis) with blue representing higher expression in non-laboring samples and red representing higher expression in laboring samples. Most of the nodes in this network have a dark blue or red color, indicating that they exhibit some differential expression between laboring and non-laboring myometrium. Multiple genes in the network upstream of our target genes have been linked to parturition or are in some of the gene sets/pathways we investigated in previous sections of this work.

The parturition network provides signaling information about quiescence and labor, or the machinery that is present in both phenotypes. To identify signaling pathways specific to each phenotype (IL vs. NL), we also constructed signaling networks specific to each phenotype. We constructed these networks by setting the input target genes as the top 50 genes associated with NL (NL network) or IL (IL network), based on their SVD gene loadings. For the remaining nodes in the network, we assigned weights to ensure that genes with expression associated with the corresponding phenotype are prioritized (i.e., have lower costs and are therefore more likely to be chosen in the shortest path). In other words, the values in the singular vectors associated with the genes were normalized such that genes up-regulated during labor had greater importance in the labor network (and vice versa for the non-labor network). The resulting networks are shown in Figure 8B (non-labor) and Figure 8C (labor). These two networks show very few similarities with each other, but both exhibit similarity with the network from Figure 8A. We also note that the networks do not contain nodes of only one color, indicating the importance of certain proteins in the signaling of both biological states. The NL network is enriched for neutrophil signaling, *EGF* receptor signaling, cAMP signaling, regulation of circadian rhythm, and *JUN* phosphorylation while the labor network shows enrichment for glucocorticoid receptor regulatory network, *TNF* signaling, signaling by interleukins, regulation of *PI3K*, and positive regulation of cell proliferation. The IL network has many downstream targets that are known to be classic inflammatory genes (*IL1B, RUNX1, PIM1, BCL3*, etc.) whereas the target genes in the NL network give us clues as to which genes may be most important during pregnancy and how they are regulated.

## Discussion

In this work, three gene expression studies derived from myometrial tissue samples of women at term (IL vs. NL) were analyzed and compared. Differential expression analysis provided a high-level examination of transcriptome variation between sample phenotypes and similarities across the three studies, which produced a high confidence set of 126 genes (differentially expressed in all studies) useful for characterizing labor. Classification through supervised machine learning algorithms LASSO and EN was used to assess the strength of the transcriptional signature within each dataset and gain insight regarding the overall quality and consistency both within and between datasets. SVD on an aggregated expression matrix further confirmed a strong transcriptional signal separating laboring and non-laboring phenotypes. Finally, an integration of the knowledge gained from these approaches and pathway analyses with network information was performed to construct a signaling network of pregnancy and labor in human myometrium.

### Overlap Between Datasets Reveals High-Confidence Transcriptional Markers of Labor

By examining multiple datasets and employing various analysis techniques, we learned more about the transcriptional landscapes of myometrial tissue at different stages of parturition as compared to previous studies that examine only one dataset using simple differential expression analysis. Investigation of the results in an integrative manner helps avoid making false conclusions or dismissing outliers of a given dataset as a consequence of the limitations for a single method. The benefit of using multiple datasets is clearly seen with the Venn diagram shown in Figure 2, where a minimum of 500 genes would be identified through any given study individually, however, upon comparison across datasets, a compact set of 126 high confidence genes is obtained. Gene expression data are inherently noisy. In this study, we have the added challenges of differing populations, study sizes, platforms, and clinical phenotypes. Therefore, the 126 identified genes meeting stringent significance criteria in all studies can be considered as a reliable signature of labor-associated genes. Included in this set of 126 genes are some well-known contraction associated proteins, including *PTGS*, *PTGES*, *COX2*, *IL8*, *CXCL2*, *CCL2*, and *SOCS3*. The oxytocin receptor (*OXTR*) is only significantly differentially expressed in the Warwick study.

### Implications of Misclassified Samples

In addition to the identification of high-confidence transcriptional markers, application of multiple computational approaches on the same data helped identify potential outliers within a given study. Namely, our comparison of misclassified samples across LASSO/EN and SVD, revealed multiple samples that showed expression patterns more consistent with the opposite phenotype (e.g., samples D-IL7 and D-IL16, which are incorrectly classified in all LASSO/EN classification tasks (training on any dataset) and scored more similarly to non-labor samples by the most dominant singular vector of SVD; the same result is seen for samples L-ILEa2, L-ILEa6, L-ILEs3 from the London dataset). Together, these results bring into question the quality of the samples as they are repeatedly misclassified in all cases. In this context, each sample has a clear phenotype distinction. However, visible characteristics of laboring women may not match the biochemical actions within the myometrium. The initial stages of labor for certain individuals may still involve further biochemical changes to achieve full activation characteristic of parturition. All three of the studies analyzed in this work adopted different clinical definitions of labor in order to designate sample phenotype. These criteria were mainly based on contraction frequency and cervical dilation (Table 1). This observation calls for a clinical standard for determining the presenting phenotype, adding to the importance of the work carried out in this study as it is possible to employ our discovered transcriptional signatures as a means to aid in phenotype calling of a given myometrial sample.

### Gene Expression Signatures Have Greater Variability in Laboring Samples

Analysis of samples via supervised learning also allowed us to draw broader conclusions about the data. One example is that the laboring phenotype has greater variability relative to the non-labor phenotype as all “questionable” samples fall into the labor class. This is not surprising as it is inherently easier for physicians to determine the non-laboring phenotype, whereas there is a broad spectrum of laboring markers (cervical dilation, frequency of contractions, etc.) There are also many factors that can influence these phenotype markers of parturition at the time of tissue sample collection, such as age, weight, race, and number of prior pregnancies.

### Data Normalization and Pre-processing

It is important to note that much of the analysis carried out in this work required an accurate procedure for creating comparable distributions of gene expression across the three transcriptome studies. It is well understood that raw data from RNA-seq and microarray platforms have dissimilar distributions and characteristics (Taroni and Greene, 2017). Therefore, to perform cross-dataset classification and SVD, a careful normalization approach was needed. There are many proposed procedures for creating agreement between RNA-seq and microarray data (Taroni and Greene, 2017). In this study, a relatively simple approach was used and was highly effective, demonstrated by the performance of our classification models and singular vectors from the SVD analysis. It is important to keep in mind that more complicated study comparisons may require other, more sophisticated techniques for sufficiently accurate results.

Other areas of consideration may also be important when combining studies in similar analysis pipelines. Here both RNA-seq studies employed paired-end sequencing and aligned around 97 percent of reads. London averaged 53 million reads per sample and Warwick averaged 7.2 million. London and Warwick were aligned and processed in the same manner in this work, leading to the identification of 23617 genes while 19897 genes were identified for the Detroit study (microarray). After removal of duplicate genes and those with very low expression, the gene expression matrices were similar in size (around 15000 genes). Finally, 410 genes are significant in both the W-IL vs. W-NL and the L-ILEs vs. L-NL, 48 of which are not tiled on the array in the Detroit study.

### Comparing Tissue Studies With Other Experimental Techniques

The expression signatures obtained in this study can also be applied to understand how well other experimental samples match to true phenotypical conditions within the myometrium, at least in terms of gene expression patterns. For example, the trained classifiers and SVD singular vectors can be employed to assess samples from new studies, cell lines, or even different uterine tissues. The similarity between cell line models and the tissues they are derived from is a very important aspect of consideration for *in vitro* research. Therefore, the consistencies and inconsistencies between tissue-based gene expression signatures and cell-line based expression signatures can serve as a means to understand the model system and assess the ability of the model system to accurately represent the biological state(s) being studied.

### Results Reveal Differences Between Computational Techniques

Our results also revealed certain strengths and weaknesses of different analysis techniques in the context of this problem. One example can be seen from the LASSO and EN classification performance on different datasets and classification tasks. We observed that models derived from the Warwick and Detroit datasets produced similar performance on the London dataset despite the fact that the number of samples in the Detroit study is much greater than that of the Warwick study. This may be simply due to the observed higher level of differential expression of the Warwick study or an artifact of comparing studies from different platforms. Additionally, the classification performance suggests that EN is better suited for cross-dataset classification. One probable reason for this is that EN, unlike LASSO, allows correlated features to be included in the final model. A LASSO model will be restricted to including only one of these correlated features, which may or may not be differentially expressed in the testing dataset. On the other hand, EN can include this gene and others with similar expression patterns. This increases the likelihood that at least one of the correlated genes will exhibit differential expression in the testing set, allowing it to improve classification accuracy. It is important to note that the genes selected by these algorithms are not necessarily the genes most associated with phenotypic changes. Rather, they represent sets of genes that can robustly distinguish different phenotypes when considered together. Many other genes may exhibit differential expression between different phenotypes, the differential expression of these gens can be more significant than that of the genes that are selected, and some of those genes may be the genes most associated with phenotypic changes. However, such genes may be left out of the model if they do not provide additional information in the presence of other differentially expressed genes. Taken together with the previously mentioned sparsity induced by LASSO and EN, it is not surprising that there was little overlap between the differentially expressed genes and those genes incorporated in the classification models. We also observed little overlap between model genes for different datasets but good cross-study classification. This likely represents the identification of different genes that are part of similar biological systems/processes. Noise in gene expression data can affect the individual dataset signatures, but these algorithms can pick up a signal present in all studies.

Another example comes from comparing the differential expression and pathway enrichment results with the SVD output. The former two analyses identified signatures that were almost exclusively associated with inflammatory processes (a large majority of the 126 shared differentially expressed genes as well as most of the significant GSVA pathways were linked to inflammation). However, SVD identified genes highly associated with the non-labor phenotype (Figure 4B), leading to improved characterization of the transcriptional programs utilized throughout parturition.

### Toward Characterizing the Systems Biology of Labor

In this work, we identified robust transcriptional signatures that could be used to allocate a clinical phenotype to a given sample. These signatures, as seen from gene and pathway enrichment analyses, were highly composed of inflammatory-related processes. This attests to the importance of inflammation in the onset of labor. However, certain pathways may be highly complex with sub-paths signaling during labor and others signaling during quiescence. This can influence analyses like pathway enrichment. For this reason, it is important, and as we showed can be very informative, to utilize network and pathway interaction information to better interpret how genes are altered in the overall scheme for a pathway of interest.

The dataset introduced in this work incorporated a new clinical group to the two existing ones (IL and NL), ILEa: early stage labor, allowing for more time points around the pivotal period of parturition. Although no significant gene expression changes were observed, it is still critical for future studies to assess biological changes during various stages of pregnancy and labor. Migale et al. (2016) carried out a temporal transcriptomics (RNA-seq) study on CD1 mice under conditions of term gestation, RU486-induced preterm labor, and lipopolysaccharide (LPS)-induced preterm labor. They identified that the LPS model (inflammatory) most closely resembled human labor, exhibiting specific changes that were also seen in this study (up-regulation of interferon, apoptosis, IL6, JAK-STAT, and cytokine signaling pathways). Salomonis et al. (2005) performed global gene expression analysis (microarray) on myometrial samples collected from FVB/N mice at multiple stages of parturition (non-pregnant, mid-gestation, late gestation, and postpartum). Results from this study exhibit strong similarity with the results presented here including upregulation of cAMP and progesterone signaling during quiescence and upregulation of contractile signaling and cell remodeling during labor. Further research in this area will help identify the timing mechanisms and responsible pathways for transitioning the quiescent myometrium to the contractile state, which involves many factors and processes (inflammatory load, functional progesterone withdrawal, tissue cross-talk) working in concert (Menon et al., 2016). Understanding these timing mechanisms and the relationship between progesterone and inflammation can then be used to model parturition and make predictions about preterm birth risk (Brubaker et al., 2016). Larger studies probing gene expression throughout pregnancy and labor in humans is much more difficult, however, steps in this direction would improve our understanding of how the uterine tissues establish and maintain quiescence, prepare for labor, and achieve parturition.

It is important to note that the transcriptome datasets were derived from total RNA extracted from whole-tissue samples, which are heterogeneous, containing myometrial cells, connective tissue and immune cells such as leukocytes. Shynlova et al. (2013) showed in mice that the number of immune cells recruited to the myometrium increases at the time of parturition. This is therefore an important consideration when assessing changes observed in the transcriptional studies analyzed in this work. To obtain a full picture of transcriptional alterations during parturition, further studies utilizing advanced experimental techniques (e.g., single-cell transcriptomics) are needed to assess gene expression changes specific to myometrial cells as well as determine the population dynamics of the non-myometrial cells during quiescence and labor.

### Translational Implications

Parturition is a complex process involving significant changes at the cellular and tissue levels. This makes it difficult to understand and treat labor disorders such as preterm labor. Understanding how parturition is carried out by the uterine tissues is key to gaining insights into the cause of labor disorders. By utilizing three large-scale molecular datasets in combination with advanced bioinformatics techniques, we have shown that there are significant changes at the level of transcription within the myometrium during the transition from quiescence to labor, identified the genes and pathways representing these changes, and constructed a signaling network of parturition. This work demonstrates the utility of bioinformatics approaches as a means to probe complex biological events, as it has provided robust transcriptional signatures that can be useful for both characterizing, by looking at the representation of these signatures in a given clinical sample, and understanding parturition. Additionally, combination of quality datasets can provide results that may not be seen through analysis of individual studies and allows for more powerful conclusions to be reached.

## Materials and Methods

### Data

This work analyzes three gene expression datasets generated from myometrial biopsies of women at term (≥38 weeks) undergoing cesarean section (Table 1). Raw data files for the Warwick dataset were downloaded from the NCBI Gene Expression Omnibus (GEO), accession number GSE50599, the Detroit study files were obtained directly from the study authors, and the London study raw data was published to GEO (GSE80172). Full details of tissue collection procedures, sample phenotyping, and mRNA expression measurement can be found in the original research papers (Mittal et al., 2010; Chan et al., 2014). The following two sections describe the methods used to generate the dataset introduced in this work.

### Sample Collection

Myometrial biopsies from women undergoing cesarean section were collected in accordance with the Declaration of Helsinki guidelines, and with approval from the local research ethics committee for Chelsea and Westminster Hospital (London, United Kingdom; Ethics No. 10/H0801/45). Informed written consent was obtained from all women who participated. Biopsies were excised from the upper margin of the incision made in the lower segment of the uterus, immediately washed with Dulbecco’s phosphate-buffered saline (Sigma) and dissected into pieces approximately measuring 2–3 mm^3^. For RNA study, biopsies were immersed in RNAlater (Sigma) within 6 min after biopsy excision from the uterus and stored at 4°C overnight, before being taken out of RNAlater solution to be frozen for long-term storage at −80°C. Labor was defined by the presence of regular uterine contractions associated with progressive cervical dilation. All specimens were categorized into three groups according to their labor stages: term not in labor (NL, *n* = 8), term in initial or early labor (ILEa, defined as cervical dilatation < 3 cm, *n* = 8) and term in established labor (ILEs, defined as cervical dilatation > 3 cm, *n* = 6). The reason NL patients underwent cesarean section included obstetric indications (e.g., persistent breech presentation) or maternal request due to tocophobia. Women in the labor group underwent cesarean section for indications including fetal distress, breech presentation and previous cesarean section. Women with gestational diabetes, preeclampsia, chorioamnionitis, multiple pregnancy, and/or given labor-augmenting drugs (prostaglandins and oxytocin) were excluded.

### RNA Extraction, Library Preparation, and Sequencing

For each sample, 60–100 mg of myometrium tissue were extracted in TRIzol (Life Technologies) by mechanical homogenisation in a Precellys 24 bead-based homogeniser using 5 cycles of 5000 rpm for 20 s, before chloroform treatment and centrifugation at 4°C. RNA was extracted from the aqueous phase of centrifuged homogenates using the TRIzol Plus RNA Purification kit (Life Technologies) with on-column DNase treatment prior to elution, all according to manufacturer’s instructions. Final RNA samples were stored at −80°C. The quantity and quality RNA was measured using a Nanodrop ND-1000 spectrophotometer (LabTech), Qubit fluorimeter (Life Technologies) and Bioanalyser 2100 (Agilent Technologies). Preparation of cDNA libraries was carried out using the TruSeq Stranded mRNA Sample Preparation kit (Illumina), following the high-throughput sample (HT) protocol. The quantity and quality of cDNA libraries were also tested by a Qubit fluorimeter and Bioanalyser 2100. TruSeq Stranded libraries were then multiplexed and sequenced with the average of 42 million DNA fragments per sample (100 bp paired-end reads). Quality control was performed using FastQC software (version 0.11.2).

### Dataset Processing

RNA-seq datasets (Warwick and London) were processed using TopHat and CuffLinks (version 2.2.1) to estimate gene expression values (Trapnell et al., 2012). For this purpose, raw RNA-seq read data, in fastq file format, were mapped to version hg19 of the human genome using TopHat to obtain sorted bam files. These bam files were then used as input for CuffLinks for transcript assembly and RNA abundance estimation. Duplicated rows and genes having zero expression in more than 3 samples were removed. The microarray dataset (Detroit; Illumina) was processed as in Mittal et al. (2010). Briefly, the raw data file was *log_2_* transformed followed by quantile normalization. Probes having expression within the background range in at least five samples were removed. Duplicate rows were also removed.

As will be described below, certain analyses in this work involved working with an expression matrix containing all samples (i.e., concatenation of the expression matrices from all studies). In these cases, additional processing and normalization was performed on the individual matrices. This is due to the differences in the RNA-seq and microarray platforms, which each have specific characteristics and expression distributions. For this reason, any classification or comparison of datasets from these different platforms required prior normalization. The two RNA-seq studies, Warwick and London, were transformed using *log*_2_*(X+1)* in order to shift them to a similar scale as the Detroit microarray study, which, as mentioned above, was already log transformed. All datasets were then row and column normalized to achieve comparable distributions of expression across genes and samples.

### Differential Expression Calculation

Warwick and London differential expression was calculated by performing CuffDiff, part of the CuffLinks software, on the output bam files from TopHat, grouping by sample type. Detroit differential expression was determined using the R (version 3.3) *limma* package by fitting linear models to the individual probes and testing significance via empirical Bayes (Smyth, 2005). Statistically significant genes were identified utilizing the *Q*-value cutoff of < 0.05 after Benjamini Hochberg procedure for multiple hypothesis correction and a fold change cutoff of 1.5.

### Classification

The R package *glmnet* was employed to perform classification with the LASSO and elastic net (EN) algorithms (Friedman et al., 2010). Classification was performed using the *cv.glmnet* function, setting the “family” argument to “binomial” for Warwick and Detroit (two sample groups: labor and non-labor) and “multinomial” for London (three sample groups: non labor, early labor, and established labor). Dataset and sample group variability were assessed by performing k-fold cross-validation on each study with *k* = 3 for Warwick and *k* = 5 for London and Detroit. A smaller *k*-value was chosen for Warwick as the study consisted of only 10 samples, while 5 was chosen for London and Detroit as 5-fold cross-validation is common practice in many classification problems. For drawing of ROC curves and calculation of AUC, the London dataset was turned into a binary problem by combining the early labor (L-ILEa) and established labor (L-ILEs) sample groups into a single laboring group whereas the multinomial method was used to calculate sample classification frequencies. Due to the variability of cross validation, one hundred rounds of training and prediction was carried out for each classification task. Model features (genes) and their coefficients were recorded for each training run. To assess the importance of each gene in discriminating labor from non-labor samples, we computed the number of models in which the gene is retained as a feature and each gene’s mean model coefficient.

To assess the reproducibility of the machine learning models, cross-classification was also performed, namely by making predictions on each of the three datasets using the model that was trained on each of the other two studies individually. This resulted in six cross-classification experiments, in which each dataset served as the training data in three experiments and as the test data in three experiments. Except for the London dataset, which was reduced to just the L-NL and L-ILEs samples for consistency with the other two studies, the full datasets were used in training, which included k-fold cross-validation to obtain optimal model parameters as calculated by the *cv.glmnet* function within the *glmnet* package. All studies were binomial classification problems and the training and prediction was performed one hundred times.

### Singular Value Decomposition

In order to extract common patterns and gene expression signatures across the three studies, we also employed SVD (Golub and Reinsch, 1970). SVD is an unsupervised mathematical approach that maps a high dimensional matrix to lower dimensional space and identifies patterns of variability within the data. For a given gene expression matrix *M* of size *mxn* (*m* genes and *n* samples), SVD decomposes this matrix into three matrices *U*, Σ, and *V*^T^ (the transpose of *V*) such that *M* = *U*Σ*V*^T^. Σ is a diagonal matrix consisting of singular values sorted from largest (first entry in matrix) to smallest (last entry). These singular values correspond to the strength of each pattern in the data (i.e., how well the pattern approximates the variability within *M*). *U* and *V* contain the left and right singular vectors where columns of *U* are considered eigen-assays and rows of *V*^T^ eigen-genes (Wall et al., 2003).

Using our normalized gene expression matrices (see previous section) for the three studies, we constructed a combined gene expression matrix *M* consisting of all 71 samples (10 Warwick + 22 London + 39 Detroit) and the 12,622 genes measured in all three studies. We then applied SVD to M. Singular values were assessed to determine the significant patterns within our expression matrix by observing the diagonal entries of Σ. The dominant gene pattern is obtained by plotting the first column of *U* and the dominant sample pattern is represented by the first row of *V*^T^.

For pathway-specific SVD analysis, the same procedure as above was performed with the difference being the expression matrix on which the analysis was done. Genes for each pathway or gene set were extracted and the gene expression matrix was reduced from the 12,622 shared, measured genes to only contain the genes involved in the pathway. The procedure was done individually for each pathway.

### Pathway Enrichment

Pathway enrichment was performed in two ways to better interpret the gene expression signatures obtained from our analyses. A high level approach was used by inputting the gene symbols of the 126 high-confidence genes, obtained from comparing differential expression across all studies (Figure 2), into the web-tool Enrichr^1^ to understand which pathways are most strongly and consistently altered between laboring and non-laboring myometrium (Chen et al., 2013). Enrichment was assessed using the KEGG 2016 result under the Pathway tab on the Enrichr results webpage (Kanehisa and Goto, 2000). Results were sorted by adjusted *p*-value.

A within-dataset approach was used by performing GSVA in *R* to identify the pathways most differentially expressed between labor and non-labor conditions specific to each dataset. This allowed for observation of consistency across datasets at a pathway level (Hänzelmann et al., 2013). The hallmark gene sets from the molecular signatures database (MSigDB; version 5.2) was obtained via the broad institute website and loaded into R using the *getGmt* function (Subramanian et al., 2005; Liberzon et al., 2015). GSVA was then applied individually to the expression matrices of the Warwick, London, and Detroit studies after IQR filtering to remove genes with low expression variance across all sample. The raw expression matrices from the Warwick and London studies were *log2* transformed and run with the argument *RNA-seq = FALSE*, as recommended in the GSVA documentation. Gene sets of size 5–500 were allowed in the enrichment analysis. Finally, the *limma* package was used to fit models to each gene set, and significance was calculated by applying an empirical Bayes statistical test (Smyth, 2005). *P*-values were adjusted using the Benjamini Hochberg procedure for multiple hypothesis testing. Clustering of the enrichment results was performed on the top 30 genes sets (selected by median *P*-value over the 4 study comparisons) using the *pheatmap R* package with correlation between fold change enrichment as the distance measure and complete clustering as the clustering method.

For the hypothesis-driven pathway analysis, MSigDB was used to identify and build a set of parturition-relevant gene sets (progesterone, inflammation, cAMP/smooth muscle) from multiple sources (GO, KEGG, etc.). GSVA was then implemented on these 32 gene sets for each study individually. Of this initial set, 26 met the 5 gene size requirement (previous paragraph) after IQR filtering. Results for all 26 gene sets were then clustered and colored by GSVA fold change values as with the global analysis.

### Annotation of Vascular Smooth Muscle Contraction Pathway Diagram

The VSMC pathway diagram was obtained from the KEGG pathway database. The pathway information was obtained by downloading the associated KEGG Markup Language (KGML) file for the “Homo sapiens (human)” option in the organism drop-down bar on the KEGG pathway website. This pathway was imported into Cytoscape version 3.3. Node (gene) colors were obtained by performing SVD on the Warwick gene expression matrix reduced to the genes in the VSMC pathway (see Singular Value Decomposition section of “Materials and Methods”). The Warwick study was used due to its observed higher quality in the classification tasks and because it allowed us to populate the greatest proportion of nodes in the pathway with a gene loading value. These gene loadings for the dominant singular vector were imported into Cytoscape as a node table and used in a continuous mapping for the node color (blue represents higher association with non-labor, red higher association with laboring samples) attribute within the “Style” tab.

### Signaling Network Construction

Parturition signaling networks were created by performing shortest path calculations on a curated signaling network from Ritz et al. (2016) (Pathlinker version 1.1). Briefly, this network was built by integrating multiple protein-protein interaction networks with pathway databases to create a high-confidence, directed signaling network. Edges in this network were weighted based on experimental evidence and commonality of gene ontology annotations. We then weighted nodes based on the gene loadings from the first singular vector obtained from applying SVD on the normalized, combined tissue expression matrix (12,622 genes × 71 tissue samples). The raw gene loadings were normalized to the range of [0,1] after performing absolute value in order to obtain appropriate node costs (nodes with cost near zero were genes with highly negative and positive singular value loadings, the differentially expressed genes, making them more likely to be chosen in the final paths).

Shortest paths were then computed between upstream, pathway initiator proteins to downstream target genes. Specifically, *Dijkstra’s* algorithm was used to calculate the shortest path between each pathway initiator and each target gene (Dijkstra, 1959). Additionally, a requirement of these shortest paths was that the last step in the path be a known transcription factor-target gene interaction (this target being one of our downstream target genes based on the gene expression studies), where transcription factor interactions were identified utilizing the TRED, TFactS, Oreganno, and TRRUST databases (Griffith et al., 2007; Jiang et al., 2007; Essaghir et al., 2010; Han et al., 2015) (combined interactions from Chouvardas et al., 2016). The pathway initiator proteins chosen here were *PGR*, *IL1R1*, and *ADCY* to represent the first protein in the signaling cascade for progesterone signaling, inflammatory signaling, and cAMP signaling, respectively, which were believed to play a role in controlling parturition through existing hypotheses and our targeted pathway analyses. *ADCY1-9* all connect to the same set of six proteins directly downstream in the signaling network, meaning that each ADCY has the same interactions or path options when building the network. Therefore, any of these ADCY nodes can be chosen as the start of the path. *ADCY9* was chosen out of these nine possible proteins due to its loading for the dominant singular vector, which was the closest in magnitude to the loadings for *PGR* and *IL1R1* than any other *ADCY*. This helps ensure that the cAMP portion of our obtained network is not unfairly diminished (increased) by a low (high) receptor node value when restricting the final network based on cost of paths.

Three separate set of genes were used to seed these shortest path calculations as the target genes. For the general parturition network, the top 50 genes associated with each phenotype based on the loading value from the first singular vector on the full tissue expression matrix were used (100 total nodes). For the non-laboring network, the top 50 genes associated with the non-labor phenotype (again, using gene values from the SVD analysis) were used as the downstream targets. Finally, the top 50 genes associated with the labor phenotype were used to create the laboring signaling network. For these latter two shortest paths calculations, the node weights of all genes in the original network were altered to favor paths with incorporated gene products related to the chosen downstream genes. In other words, for non-laboring, the raw gene loadings from SVD were again normalized to the range of [0,1] but without the prior application of absolute value. This ensured that genes highly associated with the non-labor signal have a low cost in the network while those genes associated with the labor signal have a high cost. These node weights were then reversed for the laboring network. Finally, a path cost threshold was applied to the first of these three networks to obtain a network of reasonable size (Figure 8A). For this set of target genes, the cost of each unique path (sum of node and edge weights making up path from initiator protein to target gene) was treated as a distribution, ranked from least to most costly. The final network was then obtained after discarding the top 50% of paths when sorted from highest to lowest cost.

## Data Availability

The dataset generated for this study can be found in NCBI Gene Expression Omnibus, GSE80172.

## Author Contributions

Conception, design, and execution of the RNA-seq experiment was undertaken by PL, KL, and MJ. ZS performed all data analysis, which was conceived by ZS, SM, and MK. MK supervised application of computational methods. SMsupervised biological interpretation and conceptualization of results. ZS drafted the manuscript. MK and SM edited and commented on earlier drafts. All authors read and approved the final manuscript.

## Conflict of Interest Statement

The authors declare that the research was conducted in the absence of any commercial or financial relationships that could be construed as a potential conflict of interest.

## Footnotes

1 amp.pharm.mssm.edu/Enrichr

## Supplementary Material

TABLE S1 Hundred and twenty six high-confidence gene set. Gene name, median log2(fold change), and median adjusted *p*-value for all 126 genes found to be significantly differentially expressed in the sample group comparisons W-IL vs. W-NL, D-IL vs. D-NL, L-ILEa vs. L-NL, and L-ILEs vs. L-NL.

TABLE S2 Unique genes recruited in Warwick EN models. List of genes included in at least one of the 100 Warwick EN models in 3-fold cross-validation. Column 1 provides the gene name, column 2 the percentage of model for which the gene was recruited (95 = 95 of 100 models), and column 3 the average coefficient of the gene for models in which it was recruited.

TABLE S3 Unique genes recruited to distinguish the London NL samples in LASSO models. List of genes included in at least one of the 100 London LASSO models in 5-fold cross-validation, specifically for the NL class.

TABLE S4 Unique genes recruited to distinguish the London ILEa samples in LASSO models. List of genes included in at least one of the 100 London LASSO models in 5-fold cross-validation, specifically for the ILEa class.

TABLE S5 Unique genes recruited to distinguish the London ILEs samples in LASSO models. List of genes included in at least one of the 100 London LASSO models in 5-fold cross-validation, specifically for the ILEs class.

TABLE S6 Unique genes recruited in Detroit EN models. List of genes included in at least one of the 100 Detroit EN models in 5-fold cross-validation.
