# Corrigendum: Myometrial Transcriptional Signatures of Human Parturition

**DOI:** 10.3389/fgene.2019.00515

**Published:** 2019-05-28

**Authors:** Zachary Stanfield, Pei F. Lai, Kaiyu Lei, Mark R. Johnson, Andrew M. Blanks, Roberto Romero, Mark R. Chance, Sam Mesiano, Mehmet Koyutürk

**Affiliations:** ^1^Systems Biology and Bioinformatics Program, Case Western Reserve University, Cleveland, OH, United States; ^2^Department of Nutrition, Case Western Reserve University, Cleveland, OH, United States; ^3^Imperial College Parturition Research Group, Department of Obstetrics and Gynecology, Imperial College School of Medicine, Chelsea and Westminster Hospital, London, United Kingdom; ^4^BGI Clinical Laboratories (Shenzhen) Co., Ltd, Shenzhen, China; ^5^Imperial College Parturition Research Group, Institute of Reproductive and Developmental Biology, London, United Kingdom; ^6^Cell and Developmental Biology, Clinical Sciences Research Laboratory, Division of Biomedical Sciences, Warwick Medical School, Coventry, United Kingdom; ^7^Perinatology Research Branch, NICHD, NIH, United States Department of Health and Human Services, Detroit, MI, United States; ^8^Department of Obstetrics and Gynecology, Wayne State University School of Medicine, Detroit, MI, United States; ^9^Center for Molecular Medicine and Genetics, Wayne State University School of Medicine, Detroit, MI, United States; ^10^Center for Proteomics and Bioinformatics, Case Western Reserve University, Cleveland, OH, United States; ^11^Case Comprehensive Cancer Center, Case Western Reserve University, Cleveland, OH, United States; ^12^Department of Reproductive Biology, Case Western Reserve University, Cleveland, OH, United States; ^13^Department of Obstetrics and Gynecology, University Hospitals of Cleveland, Case Western Reserve University, Cleveland, OH, United States; ^14^Department of Electrical Engineering and Computer Science, Case School of Engineering, Case Western Reserve University, Cleveland, OH, United States

**Keywords:** parturition, myometrium, gene expression networks, classification, inflammation

“Pei F. Lai” and “Kaiyu Lei” were not included as authors in the published article. Due to the addition of authors, the list of affiliations had been updated accordingly. The corrected Author Contributions Statement appears below.

